# S14-4 The unique role of community sport motivators in ‘connecting the dots’ in the Netherlands

**DOI:** 10.1093/eurpub/ckad133.070

**Published:** 2023-09-11

**Authors:** Lonneke Schijvens, Willemijn van Aggelen, Jacqueline Kronenburg

**Affiliations:** Knowledge Centre for Sport & Physical Activity, The Netherlands; Buurtsportcoach, The Netherlands; Knowledge Centre for Sport & Physical Activity, The Netherlands

## Abstract

**Purpose:**

In 98 % of all municipalities in the Netherlands, community sport motivators (in Dutch: Buurtsportcoaches) connect the dots between the sports sector, social sector, schools, care and childcare and even more. Their goal: motivating people of all ages to take up sport or become physically more active.

**Project description:**

With now almost 6.000 of these professionals, their role is vital in Dutch sport policy and implementation, with tasks such as physical activity education, organizing events and activities, and advise clubs and childcare organizations. They work with local and social organizations and strengthen intersectoral collaboration. The implementation is coordinated on a national and local level and the co-funding is innovative: the government funds 40%, municipalities or local sport organisations 60%. The role of these professionals has been defined in national policies. One of the success factors is the fact that the tasks can be locally customized, according to the needs on a local level.

Evaluation has been performed with quantitative and qualitative research. For instance, research shows that community sports coaches are responsible for both organising (95%) and supervising (86%) extracurricular sports activities at schools. A large majority (82%) of municipalities want to use community sports coaches to ensure that more children meet the exercise standard and that their motor skills improve.

**Conclusions:**

Community sports motivators are here to stay in the Netherlands. Long-term sport policy is needed to build up the confidence in and capacities of this new sport professional. Their role is evolving: in the new National Sports Agreement (2022), strengthening implementation power is one of the targets. Community sports motivators will be more often deployed to organise or realise the referral of inactive people to the existing offer. And with the new School & Environment programme, the Dutch government is committed to equal opportunities by giving sufficient sports and exercise an extra impulse. This starts in the neighbourhoods where the most vulnerable children live, also lagging behind in sports and exercise participation. Among started schools, good examples are already visible where clubs, community sports coaches and commercial sport providers make an important contribution to children's development.

